# Long-term refined genomic analysis of tuberculosis clusters to distinguish between ongoing transmission, reactivations or diagnostic delays, Almería, Spain, 2003 to 2024

**DOI:** 10.2807/1560-7917.ES.2026.31.11.2500301

**Published:** 2026-03-19

**Authors:** Cristina Rodríguez-Grande, Silvia Vallejo-Godoy, Miguel Martínez-Lirola, Sheri M. Saleeb, Marta Herranz, Sergio Buenestado-Serrano, Andrea Marcos-Abellán, Pilar Barroso-García, Senay Rueda Nieto, Francisca Escabias-Machuca, Ana Belén Esteban-García, María Teresa Cabezas Fernández, José Antonio Garrido-Cárdenas, Patricia Muñoz, Laura Pérez-Lago, Darío García de Viedma

**Affiliations:** 1 Servicio de Microbiología Clínica y Enfermedades Infecciosas, Hospital General Universitario Gregorio Marañón, Instituto de Investigación Sanitaria Gregorio Marañón (IiSGM), Madrid, Spain; 2 Servicio de Medicina Preventiva, Vigilancia Epidemiológica y Salud Pública, Hospital Universitario Poniente, Almería, Spain; 3 Departamento de Microbiología, Complejo Universitario Hospitalario Torrecárdenas, Almería, Spain; 4 CIBER Enfermedades Respiratorias (CIBERES), Madrid, Spain; 5 Epidemiología. Distrito Sanitario Almería, Spain; 6 Epidemiología. AGS Norte de Almería, Almería, Spain; 7 Servicio de Análisis de Ácidos Nucleicos, Servicios Centrales de Investigación de la Universidad de Almería, Almería, Spain; 8 Departamento de Biología y Geología, Universidad de Almería, Almería, Spain; 9 Departamento de Medicina, Universidad Complutense de Madrid, Spain; * These authors contributed equally to the work and share last authorship.

**Keywords:** Tuberculosis, genomics, epidemiology, transmission

## Abstract

**BACKGROUND:**

In tuberculosis (TB) surveillance, genomics is mainly used to identify TB patient clusters; growing clusters are commonly attributed to ongoing transmission events.

**AIM:**

This study’s objective was to explore other factors, in addition to ongoing transmission, contributing to cluster expansion.

**METHODS:**

The study population included all 1,886 culture-positive TB cases diagnosed within the whole Almería province population, Spain, between January 2003 and June 2024. Cases’ *Mycobacterium tuberculosis *strains were whole genome sequenced enabling detection of clusters (with pairwise distance between strains < 12 single nucleotide polymorphisms (SNPs)). Evolutionary analyses positioned cases within genomic networks based on SNP distribution. This allowed, together with clinical and epidemiological data, to infer why new cases (diagnosed 3.5 years prior) entered clusters.

**RESULTS:**

Cases’ mean age was 37.3 years (standard deviation: 16.4); 71.7% (1,352/1,886) were male and 65.2% (1,230/1,886) migrants from 50 countries, with mostly Moroccan (21.6%; 407/1,886), Romanian (10%; 188/1,886), Senegalese (8.3%; 156/1,886) and Malian (5.2%; 98/1,886) nationalities. We detected 106 clusters, comprising 537 cases in total. The 106 new cases occurred within 53 clusters, including 31 growing clusters (identified pre-2021) and 22 recent clusters (that arose in 2021 and after). Ongoing transmission was responsible for cluster expansion in around one-third of growing clusters (9/31), versus two-thirds (15/22) of recent clusters. Genomic network assessments found that newly clustered cases not due to ongoing transmission, were likely driven by reactivation of past exposures, prolonged diagnostic delays or subclinical periods, or a combination of these factors.

**CONCLUSION:**

Understanding cluster dynamics guides case-specific management and supports TB control.

**Key public health message**

**What did you want to address in this study and why?**

In tuberculosis (TB) surveillance, genomics serves to identify patient clusters, i.e. groups of patients infected with very similar strains of *Mycobacterium tuberculosis*. With time, more patients can be assigned to a cluster, causing its growth. Cluster growth is often attributed to ongoing TB transmission. To improve TB control, we used an evolutionary approach to see if other additional factors could cause cluster growth.

**What have we learnt from this study?**

Genomic evolutionary analysis, combined with clinical and epidemiological data, revealed that most growing TB clusters were not caused by ongoing transmission events but by reactivations of old infections or new diagnoses in patients who had been temporarily living with undiagnosed or subclinical TB. Understanding underlying cluster dynamics is critical to guide appropriate measures to address TB spread.

**What are the implications of your findings for public health?**

Identifying whether a TB case is due to ongoing transmission, reactivation, or delayed diagnosis allows for tailored responses. These include expanding and reinforcing contact tracing, optimising preventive treatment and identifying non-diagnosed cases mainly in clusters growing due to recent transmission, but also in clusters growing due to reactivations. In diagnostic delay events, secondary cases must be actively searched.

## Introduction

Whole genome sequencing (WGS) of *Mycobacterium tuberculosis* (MTB) has revolutionised the way we study the transmission dynamics of tuberculosis (TB) among people. Since genomic thresholds (i.e. pairwise distance between strains infecting patients of < 12 single nucleotide polymorphisms (SNPs)) have been defined for clusters of TB patients [1], and for likely recent transmission events (< 5 SNPs [2]) among patients in clusters, these criteria have been applied in many settings of the world [3-5].

In standard genomic epidemiology programmes, analysing SNPs in MTB strains is frequently conducted to identify clusters of TB patients. Finding these clusters sheds light on the epidemiological contexts where ongoing TB transmission occurred. Knowing such contexts enables to adapt control interventions accordingly to prevent new secondary cases. While the use of SNPs to delineate clusters is important, it should be noted that additional valuable epidemiological information can be derived from SNPs arising within the cluster. Indeed, a detailed assessment of how differential SNPs are distributed among patients in a cluster may allow to infer the transmission chronology, or the most likely person-to-person transmission relationships [6].

In a socio-epidemiologically complex population, this extra information can be particularly helpful to investigate the dynamics of TB transmission, which can be particularly intricate and require control measures tailored to each situation to succeed. Such a complex population exists in Almería, a south-eastern Spanish province, where between 2003 and 2024, 67.1% of all TB cases were migrants from different countries and TB incidence rates were four to five times higher than in the rest of Spain [7].

To optimise epidemiological TB control interventions and to improve the use of limited resources to address transmission hotspots, this study aimed to analyse detailed genomic data within TB clusters that occurred in Almería province in the past 21 years (2003–2024), to find the most likely reasons for the recent growth of active clusters. 

## Methods

### Study design and general scheme of analysis

#### Study setting 

This study is part of a research project initiated in 2003, which is linked to the Almería TB prevention and control programme (TBPCP). The TBPCP is integrated in the Epidemiological Surveillance System of Andalusia (SVEA), an extensive surveillance system within the Spanish Epidemiological Surveillance Network (RENAVE). The Almería TBPCP involves a multidisciplinary team including epidemiologists, microbiologists, professionals in preventive medicine units, nurses, social workers, as well as professionals in primary care centres and in the three hospital TB clinical units of the three respective health districts of the province. In 2003, community health workers were also incorporated into the programme, through collaboration agreements with local non-governmental organisations (NGOs) present in the territory (Red Cross and Consortium of Entities for Comprehensive Action with Migrants). The role of these community health workers is to act as cultural mediators and translators, during follow-up of TB cases and contact tracing, to support the public health system.

#### Study population

The reference population for the study consisted of all 750,000 inhabitants of the three health districts of the province of Almería, Spain. As part of the national healthcare system, all province residents are provided with universal healthcare coverage. All patients (January 2003–June 2024) among the whole province inhabitants (both migrants and autochthonous) who were diagnosed with culture-confirmed TB, under follow-up by the three hospitals’ TB clinical units and notified to SVEA following the processes in place for reporting notifiable diseases were included in the study population.

#### Data collection

To register the main characteristics of the most relevant clusters identified in the study, a data collection form was designed for the involved TB cases. The aim was to understand the transmission environments, as well as to gain knowledge about certain risk factors that have been prior identified in past molecular and genomic research projects conducted by our research group in Almería (data not shown). In addition, data at the individual level were obtained from the SVEA registries, which store case-based epidemiological information. Registries of SVEA combine hospital data from preventive medicine units and community data from primary care epidemiology units. The data are validated and contrasted by experienced epidemiologists. For the cases in clusters which required more in-depth analyses, the patients’ clinical records were also consulted.

#### Molecular and genomic tandem analysis

The analysis of the MTB isolates in Almería was based on a two-step molecular/genomic analysis. For each smear-positive TB case, mycobacterial interspersed repetitive units-variable number of tandem repeats (MIRU-VNTR) analysis was performed directly on clinical samples, allowing to exclude non-clustered orphan cases. Isolates included in MIRU-defined clusters were then sequenced using standard Illumina approaches; clusters were confirmed when < 12 SNPs were identified between the isolates’ sequences [1]. Isolates that were considered as belonging to orphan cases by MIRU-VNTR were kept for forthcoming comparisons, in the event that any future case would form a cluster with one of these (until then) considered orphans.

We differentiated between (i) recent clusters, which were those identified for the first time in the last 3.5 years (2021–June 2024), and (ii) growing clusters, which had been previously identified in the period 2003–2020 and incorporating new cases in the last 3.5 years.

### MIRU-VNTR analysis

For MIRU-VNTR, DNA was purified from the clinical sample after heat inactivation using the GXT NA Extraction Kit (Hain Lifescience, Nehren, Germany) according to the manufacturer's instructions.

The 24-MIRU VNTR typing was performed on purified DNA from clinical samples as previously described [8], except for the final PCR volume (20 µL for each reaction) and the number of PCR cycles (45 cycles). Briefly, PCR was performed using eight triplex PCR reactions. The standard protocol was followed using the multiplex PCR kit (Qiagen) for mixes 1, 2, 3, 5, 6 and 8 [9]. For mixes 4 and 7, PuReTaq Ready-To-Go PCR beads (GE Healthcare, Chicago, United States (US)) were used with 0.5 mM of each primer and 3% dimethyl sulfoxide. If there was insufficient amplification for any locus, simplex PCR was conducted. Fragments from PCR were sized by capillary electrophoresis in a 3500 Genetic Analyzer (Thermo Fisher Scientific, Waltham, US). The MIRU-VNTR alleles were assigned using GeneMapper v5 (Thermo Fisher Scientific, Waltham, US). The MIRU-VNTR based clusters were assigned for identical patterns, tolerating only one single locus variant.

### Whole genome sequencing

For sequencing, DNA was purified from Mycobacteria Growth Indicator Tube (MGIT) subcultures using the QIAamp DNA mini kit (Qiagen, Courtaboeuf, France) according to the manufacturer's instructions, after boiling for 10 min and pre-incubation with proteinase K (20 mg/mL) at 56°C overnight.

Libraries for cluster confirmation by WGS were prepared using the Nextera XT kit (Illumina, San Diego, US) according to the manufacturer's instructions and pooled for sequencing on a MiSeq or Nextseq instrument (2 × 151 bp).

Sequence analysis was performed using an in-house pipeline deposited on GitHub: https://github.com/MG-IiSGM/autosnippy. The workflow of this pipeline follows the same steps as previously described [10], using a hypothetical MTB ancestral genome [11] as a reference. Finally, genomic distances between sequences were calculated using Jaccard similarity and Hamming distance metrics to generate distance matrices. Alignments and SNP variants were visualised and checked using the Integrative Genomics Viewer (IGV) programme.

### Genomic-based proposal of the likely reasons behind clustered cases

We proposed the most likely reasons for the clustering of cases in the most recent study period, (2021–June 2024), based on the detailed analysis of genomic data. Once the newly clustered cases were identified, we obtained the genomic networks (Network (5.0, 10.0) or PopART 1.7) for each of the clusters involved by placing the cases in the same network according to the differential SNPs identified between their sequences.

Taking into account the rate of acquisition of SNPs in MTB (1 SNP every 2–3 years), we considered ongoing transmission as the reason for a new case entering a cluster if the new case was located in the genomic network close (at 0–2 SNPs) to another case diagnosed 0–3 years before.

For the cases entering a cluster without fulfilling the previous criteria, reasons other than recent ongoing transmission were considered. Among them, cases entering clusters, which located in their networks close (0–1 SNP) to other cases diagnosed ≥ 4 years before were considered to potentially result from the reactivation of a past exposure. Finally, for the cases entering clusters with a higher number of SNPs relative to the cases preceding them in the network, we considered the involvement of a certain diagnostic delay/subclinical TB, responsible for a viable bacteria acquiring those intermediate SNPs before diagnosis, probably due to intra-host evolution.

As the first step of assigning likely reasons behind clustered cases meant to determine the chronology between the case under study and the preceding case in the network, all cases entering into clusters which were preceded by a non-sampled node (an evolutionary node not occupied by any diagnosed case) could not be considered for the analysis.

Finally, the interpretations based on genomic data were discussed, to evaluate whether they could be validated with the clinical/epidemiological data, in weekly online meetings involving the microbiologists responsible for the diagnostic tasks, the epidemiologists, and the staff responsible for the genomic analysis, with periodic support from the clinicians managing the cases as required.

## Results

### General analysis of clusters

During the study period (January 2003–June 2024), 1,886 culture-positive TB cases were diagnosed in Almería. The mean age was 37.3 years (standard deviation (SD): 16.4), 71.7% (n = 1,352) were males, 28.3% (n = 534) were females; 65.2% (n = 1,230) were migrants from 50 different countries, with the most represented nationalities being Moroccan (n = 407; 21.6% of the total cases), Romanian (n = 188; 10%), Senegalese (n = 156; 8.3%) and Malian (n = 98; 5.2%). For 90.6% of the cases (1,709/1,886) MIRU-VNTR was performed. This assigned 984 of the 1,709 cases (57.6%) to 126 clusters. Among these 126 clusters, 106 clusters (84.1%), which included a total of 615 cases, had genomic data available, with 537 genomically confirmed cases in the 106 clusters (2–25 cases/cluster).

Our first aim was to analyse the likely reasons for the clustering of cases in the most recent study period, January 2021–June 2024. Of the 306 total new cases diagnosed in this period 106 (34.6%) were included in 53 clusters (< 12 SNPs) as described in Supplementary Figures 1 and 2, Supplementary Table 1 and 2. Of these, 46 cases (43.4%; 46/106) formed 22 clusters (2–6 cases/cluster), considered as recent clusters, identified for the first time in the last 3.5 years. The remaining 60 (56.6%; 60/106) corresponded to cases entering in 31 clusters, previously identified in the period 2003–2020, considered as growing clusters, now incorporating 1–11 new cases.

Once the newly clustered cases were identified, the genomic networks were analysed to find the most reasonable explanation for why the new cases contributed to cluster growth and to recommend the most appropriate interventions for each case.

### New clustered cases due to ongoing transmission

**Figure 1:**
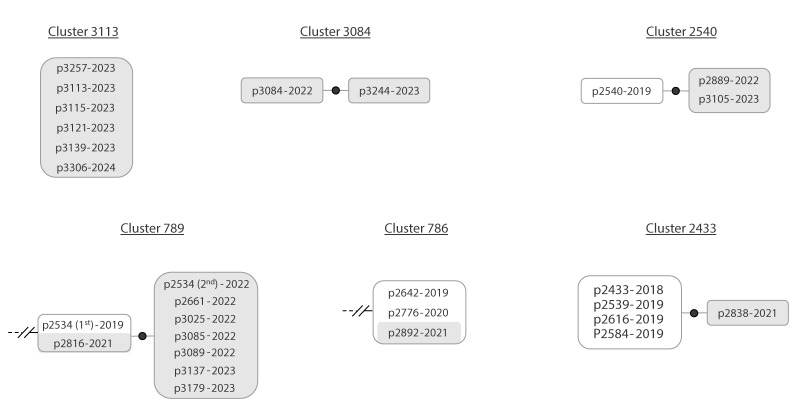
Examples of clusters with new cases which are candidates for having acquired TB as a result of ongoing transmission, Almería, Spain, 2003–2023 (n = 6 clusters)

Considering the rate of acquisition of SNPs in MTB (1 SNP every 2–3 years), we considered ongoing transmission as the reason for a new case entering a cluster if the new case was located in the genomic network close (at 0–2 SNPs) to a recently diagnosed case (0–3 years before; as exemplified in Figure 1). This criterion was met in 47.2% (50/106) of the new clustered cases and 23 of the 53 total clusters, which are illustrated in Supplementary Figure 1.

TB: tuberculosis.

Cases diagnosed in 2021–2024 and classified as likely resulting from ongoing transmission are shaded. The first number corresponds to the case and the second to the year of diagnosis. In the genomic networks, black dots represent SNPs and cases within the same box correspond to identical strains (0 SNPs between them). As indicated by the dashed lines, clusters 789 and 786 represent only a part of a larger pre-existing cluster. ‘1^st^’ and ‘2^nd^’ refer to two TB episodes experienced by patient 2534 in cluster 789.

Focusing on the 22 recent clusters (Supplementary Figure 1), which are listed in Supplementary Table 1, around two-thirds of them (68.2%; 15/22) likely grew due to ongoing transmission. One of these clusters corresponded to an extensive transmission involving six new cases (Figure 1, cluster 3113), while the others were more limited, including only one with four new clustered cases (Supplementary Figure 1, cluster 3133) and the rest with one to two new cases. 

When we focused on the 31 clusters already defined before 2021 but still growing in the last 3.5 years (Supplementary Figure 1, listed in Supplementary Table 1), ongoing transmission was identified as the reason for their growth in only nine of them, corresponding to approximatively one-third (9/31) of the growing clusters (Figure 1, clusters 789 and 786). Among the growing clusters with ongoing transmission, two had a high number of new cases (Figure 1, cluster 789: eight cases; Supplementary Figure 1, cluster 558: five cases) while and the remaining clusters had only one new clustered case.

When the genomic network interpretation proposed cluster growth to be likely due to ongoing transmission, the epidemiological data were reviewed (cases were reinterviewed when necessary) to confirm the potential relationships between cases. Moreover, to mitigate the spread of TB, the recommended epidemiological intervention was to expand the study of the patient's context to (i) identify new infected contact cases and thus increase the number of preventive treatments and (ii) identify undiagnosed active cases. From 2021 to June 2024, we identified 17 secondary cases from seven different clusters (015, 2540, 630, 789, 3083, 3113 and 3133) that had not been diagnosed before this intervention. In a further 11 new cluster cases, genomic data led to reorientate the ongoing epidemiological investigation towards settings that had not been considered before, to enhance surveillance for new cases.

#### Impact of the COVID-19 pandemic on ongoing TB transmission

Among the clusters from which the genomic network analysis indicated ongoing transmission, we must highlight one example whose characteristics helped us to assess the impact of the COVID-19 pandemic on TB transmission. The cluster (Figure 1, cluster 789) involved a case (p2534) with two TB episodes (one in 2019, before the pandemic, and the other in 2022) due to lack of adherence to treatment. The strain isolated from the first and second episodes differed in 1 SNP. This SNP was used as a marker SNP to determine the number of new secondary cases due to exposure to case p2534 either before (lacking the differential SNP) or during the pandemic (harbouring the SNP acquired in p2534 second episode). Only one new case (case 2816) diagnosed in 2021 was found to have the same sequence as case 2534’s first episode (lacking the SNP), while six cases diagnosed in 2022–23 were identical to case 2534’s second episode (harbouring the SNP). Notably, five of the six within-pandemic secondary cases had had household contact with case 2534 at some point. These findings strongly suggest that factors associated to the pandemic (the lockdown and the delayed diagnosis of new secondary cases) were key drivers of this transmission event, by impacting on the emergence of secondary cases: the exposure to the same index case, before or during the pandemic, led to a rather different number of secondary cases.

### Reasons other than ongoing transmission behind new clustered cases

The position in the genomic networks of new cases entering seven of the 22 recent clusters and 22 of the 31 growing clusters, was less consistent with ongoing transmission. By examining the positions together with the networks’ general topology, we could propose for some of them other likely alternative explanations for their clustering. These likely explanations are further presented together with the cases in question without differentiating between new cases entering recent or growing clusters.

#### Reactivations of previous exposures

**Figure 2:**
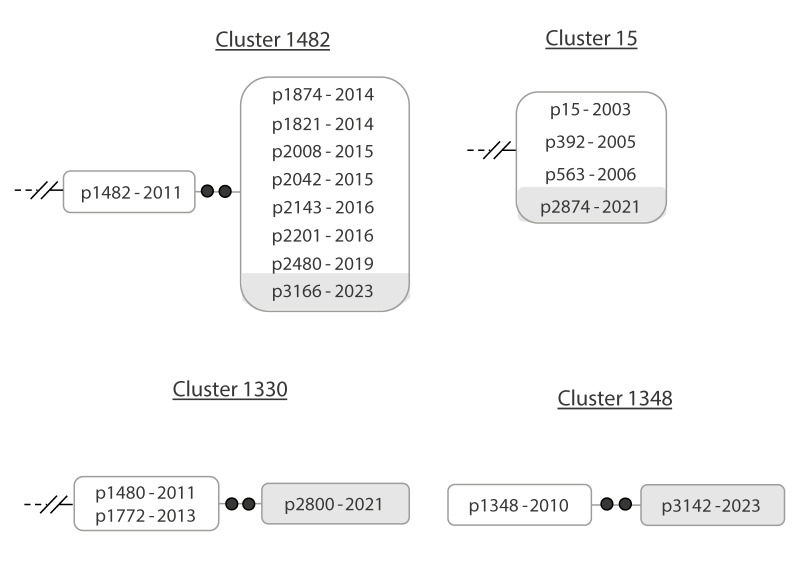
Examples of clusters representative of the involvement of cases candidates for TB reactivations, (clusters 1482 and 15) and reactivations with a certain period of diagnostic delay (clusters 1330 and 1348), Almería, Spain, 2003–2023 (n = 4 clusters)

Ten of the cases, entering 10 clusters, located in their networks close (0–1 SNP) to other cases diagnosed ≥ 4 years before (Supplementary Figure 1). These findings may suggest that these new TB cases in the clusters were due to likely reactivation of a past exposure. Figure 2 illustrates this for clusters 1482 and 15.

TB: tuberculosis.

Cases diagnosed in 2021–2024 and which were deemed as candidate cases for reactivation are shaded. The first number corresponds to the case, the second to the year of diagnosis. In the genomic networks, black dots represent SNPs and cases within the same box correspond to identical strains (0 SNPs between them). As indicated by the dashed lines, clusters 1482, 15 and 1330 represent only a part of a larger pre-existing cluster.

In addition, we identified another six clusters in which new cases also entered the network after a case diagnosed ≥ 4 years before, also suggesting the likely involvement of reactivations in this cluster; however, unlike the previous examples, we now detected some more SNPs (2–3 SNPs) with respect to the preceding case in the network (Supplementary Figure 1). Figure 2 provides examples of two of these six clusters, namely clusters 1330 and 1348. For these new cases, a possible explanation for the intermediate SNPs was the involvement of a certain diagnostic delay in the cluster, in addition to the likely reactivation. This delay would have been responsible for a period of bacterial viability before the diagnosis of the clustered case, probably due to intra-host evolution leading to the acquisition of SNP diversity.

Having identified the clusters in which reactivations were likely to have underpinned growth, we looked for data supporting this hypothesis in the epidemiological and clinical records of the cases involved and discussed the findings in our multidisciplinary team. We found reasons which could support the likely involvement of reactivation in four of the sixteen clusters with genomic findings suggesting reactivation (cases 3149, 3002, 3081 and 3184 from clusters 1202, 558, 143 and 1180, respectively, Supplementary Figure 1). Case 3149, which had epidemiological links with the two preceding TB cases in the same cluster 1202 in 2015 (Supplementary Figure 1), had abandoned diabetes treatment 2 years before TB diagnosis in 2023 and had also been hospitalised for severe COVID-19 in 2023. Case 3002 had discontinued HIV treatment before being diagnosed with TB in 2022, when a worsening of immunosuppression occurred. Case 3081 which was diagnosed in 2022, and a close contact 5 years earlier of the preceding case in the network (case 2327) in cluster 143, did not receive preventive treatment for TB and was diagnosed as a person living with HIV just before the TB diagnosis. Finally, in cluster 1180, case 3184, a close contact 5 years earlier of the preceding case 2295, did not receive preventive treatment, had poorly controlled diabetes and was found with an increased viral load of hepatitis C virus before TB diagnosis.

#### Prolonged diagnostic delay/subclinical TB

**Figure 3:**
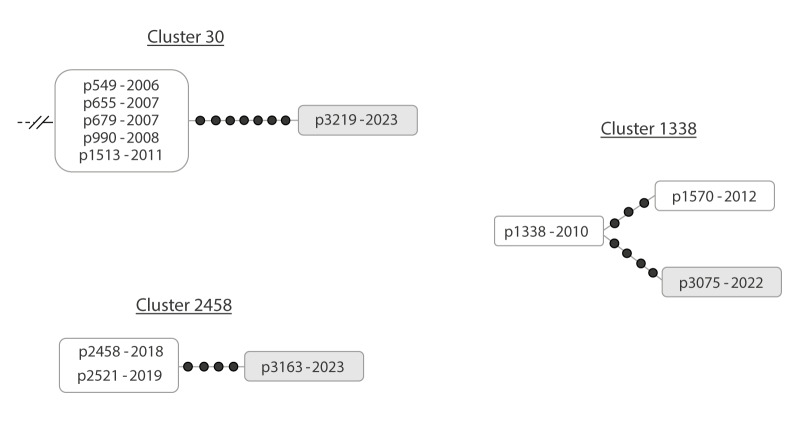
Examples of clusters containing cases that are candidates for prolonged diagnostic delay or subclinical TB, Almería, Spain, 2003–2023 (n = 3 clusters)

In eight other new cases, the number of intermediate SNPs between them and their preceding cases in the network was higher (4–8 SNPs). In terms of the rate of SNP acquisition in MTB, the number of intermediate SNPs and the difference between the time of TB diagnosis of these new cases and the respective preceding cases were compatible with the mutation rate (Supplementary Figure 1). This suggested the acquisition by MTB of successive SNPs during the whole period between the newly diagnosed case and the preceding cases. We considered these new cases as patients who probably experienced a prolonged diagnostic delay or subclinical TB (Figure 3).

TB: tuberculosis.

New cases which are patients who likely experienced a prolonged diagnostic delay or subclinical TB are highlighted by shading. The first number corresponds to the case, the second to the year of diagnosis. In the genomic networks, black dots represent SNPs and cases within the same box correspond to identical strains (0 SNPs between them). As indicated by the dashed lines, cluster 30 represents only a part of a larger pre-existing cluster.

Our candidates for likely prolonged diagnostic delay underwent clinical/epidemiological review within our interdisciplinary team to assess whether we could find some support for this hypothesis in some of them. In two of the candidate cases for prolonged diagnostic delay, we found robust evidence (Figure 3, cases 3219 and 3163 from clusters 30 and 2458, respectively). Case 3219 had radiological findings compatible with TB 5 years before TB diagnosis and had had asthenia since these findings. For case 3163, 5 years before TB diagnosis, radiology which had been performed following a tuberculin-positive result had already yielded findings compatible with TB; while this patient was referred, at the time of these findings, to pneumology, the case did not attend until final diagnosis in 2023, secondary to other procedures. Non-specific symptoms were found in the clinical history of this case over the previous 5 years.

#### Impact of diagnostic delay on secondary cases

During the time that cases with a diagnostic delay were undiagnosed, they could have resulted in people being unknowingly exposed to TB. We found three examples that allowed us to assess a progressive impact on the number of secondary cases, resulting in (i) no secondary cases, (ii) limited exposure resulting in one secondary case within the household, and (iii) several potential secondary cases in the community. These examples are described as follows.

In the first example (Figure 3, case 3219 in cluster 30), despite a long diagnostic delay, no secondary cases were found, as indicated by the absence of other cases in the genomic network along the branch leading up to this case, i.e. during the period of intra-host evolution of the strain prior to diagnosis. A review of the case within the interdisciplinary team revealed that this individual had an extremely low level of social interaction, which may explain the lack of further transmission.

**Figure 4:**
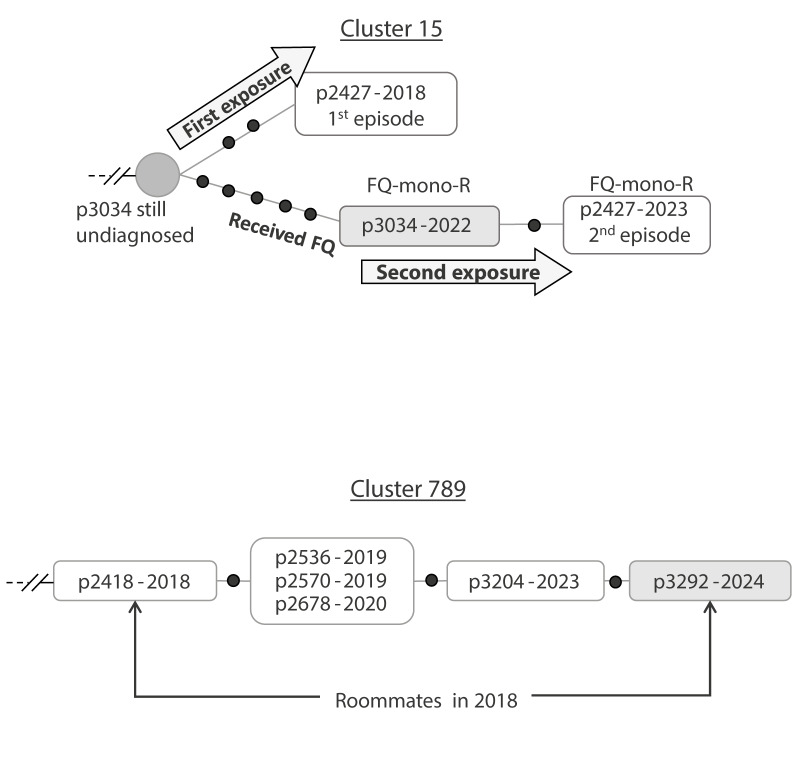
Impact of diagnostic delay/subclinical TB on transmission patterns in representative clusters from Almería, Spain, 2003–2023 (n = 2 clusters)

FQ-mono-R: fluoroquinolone monoresistance; TB: tuberculosis.

The second example (Figure 4, cluster 15) appeared to correspond to a prolonged diagnostic delay (in case 3034) potentially resulting in the exposure of a member of the same household on two occasions. Case 3034 had two features supporting a prolonged diagnostic delay: (i) several inferred SNPs prior to diagnosis in the branch of the genomic network in which the cases was located, and (ii) fluoroquinolone monoresistance (FQ-mono-R) at diagnosis. Case 3034 had previously received this drug on several occasions to treat a long-term renal/urinary condition; by receiving this drug before TB was diagnosed, monotherapy could have occurred, leading to the acquisition of resistance in the strain responsible for TB. From the analysis of the network, the most likely explanation is that case 2427 was exposed to case 3034 twice, once at a more recent occasion, explaining the FQ-mono-R in the 2023 isolate of case 2427, and the other at an earlier time, during the prolonged diagnostic delay of case 3034, which resulted in case 2427's first infection in 2018, before case 3034 acquired the FQ-mono-R.

Cases suspected of prolonged diagnostic delay or subclinical TB (highlighted as shaded) have different effects in terms of resulting in secondary cases. Cluster 15 illustrates limited sequential household exposures in one secondary case, while cluster 789 shows potentially higher numbers of exposures and secondary cases in the community, likely resulting from one case, which remained undiagnosed during several years. In cluster 15, the grey node (grey circle) corresponds to a hypothetical node occupied by the case which transmitted TB to another member of the household. This node represents the infecting strain of the case before TB diagnosis and prior to both the first transmission of TB to the secondary case in the household and the strain’s acquisition of fluoroquinolone monoresistance.

In the diagrams, the first number corresponds to the case, the second to the year of diagnosis. In the genomic networks, black dots represent SNPs and cases within the same box correspond to identical strains (0 SNPs between them). As indicated by the dashed lines, clusters 15 and 789 represent only a part of a larger pre-existing cluster.

Interpreting the impact of the last example of prolonged diagnostic delay (case 3292) on secondary cases was more complex, as the initially prolonged undiagnosed case did not have, upon diagnosis in 2024, multiple SNPs separating it from the previous case in the network for the same cluster (Figure 4, cluster 789). For case 3292, clues about potential exposures came from clinical and epidemiological information. Case 3292 had presented at diagnosis with a clinical status suggestive of long-term evolved TB, namely severe weight loss over the last few years and extensive lung damage. This case was the roommate of case 2418 in 2018. While case 2418 was in the same cluster as case 3292, case 2418 was not the case immediately preceding case 3292 in the genomic network. Indeed, case 2418 had been diagnosed with TB in 2018, 6 years before the diagnosis of case 3292. At the time of case 2418 diagnosis, case 3292 which was the roommate, had declined participating in the epidemiological investigation. Moreover, until diagnosis in 2024, case 3292 had not attended any healthcare centre. Therefore, although case 3292 was at the end of a branch in the genomic network, this position was not the result of exposure to the preceding cases in the cluster, but more likely from TB acquired from case 2418 around 2018. Since then, the strain in case 3292 may have evolved due to the prolonged diagnostic delay. This means that other cases in the cluster that included cases 2418 and 3292 (Figure 4), namely cases 2536, 2570, 2678 and 3204 could be considered as secondary cases of direct/indirect exposure to case 3292, while the MTB in case 3292 progressively gained 3 SNPs along the branch that separates it from case 2418 and that connects also all the other cases. An epidemiological investigation based on this hypothesis showed that all cases involved in the cluster lived in the same neighbourhood and shared common activities.

### Clusters growing through several factors

**Figure 5:**
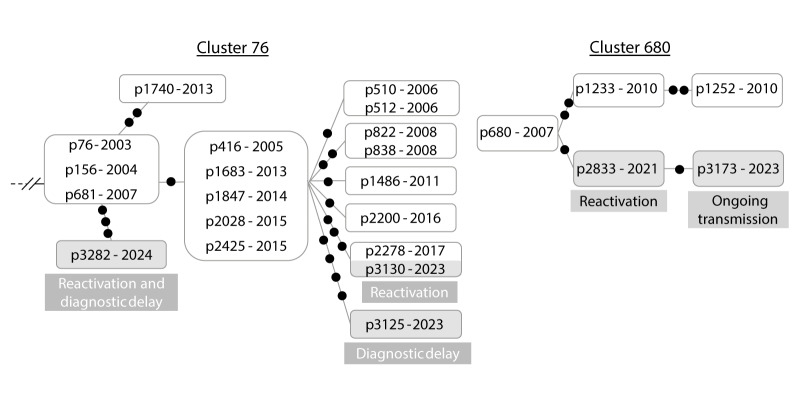
Examples of clusters expansions involving several factors, with newly clustered cases caused by ongoing transmission, reactivation, or diagnostic delay, Almería, Spain, 2003–2023 (n = 2 clusters)

SNP: single nucleotide polymorphism; TB: tuberculosis.

In six clusters, we observed the entrance of several new clustered cases, for the period 2021 to 2024. Each of these cases could have different explanations among those previously presented as reasons for cluster growth, i.e. ongoing transmission, reactivations or diagnostic delays (Figure 5).

The first number corresponds to the case and the second to the year of diagnosis. In the genomic networks, black dots represent SNPs, and cases within the same box correspond to identical strains (0 SNPs between them). Newly clustered cases are shaded.

## Discussion

Compared to traditional molecular methods, WGS improves the delineation of MTB transmission clusters. Previous studies have also noted the high accuracy of this tool to infer transmission, making it key for epidemiological research in TB [12]. 

In standard populations, the first output of any genomic epidemiology study of TB is the assessment of the proportion of clustered cases, based on SNP thresholds already validated to infer transmission or even very recent transmission [1]. This allows providing a snapshot of TB transmission patterns and dynamics in these populations and enables evaluating the effectiveness of TB control programmes. To illustrate this, among many other studies, rates of very recent transmission of 43.7% were identified in southern Brazil between 2011 and 2014 [3,13].

In socio-epidemiologically complex populations, however, the TB prevalence may be due to multiple causes. Such is the case for the population of the province of Almería in south-eastern Spain, where we conducted our research. In Almería, the TB rate is particularly high among migrants (80% in 2023), as has been reported in other similar settings where a concentration of TB cases can occur in this vulnerable group [14]. Previous findings by our team [4] demonstrate the overlap of imported TB cases with those acquiring TB after arrival, the existence of transmission between cases of different nationalities and, finally, reactivations of TB triggered by substandard living conditions of the migrant population.

All these factors justify the need for a deeper understanding of the reasons behind each new TB diagnosis. Thus, this study exploited genomic data beyond the quantitative determination of the number of SNPs between cases, which is used to rule in or rule out clustering, by also investigating the evolutionary patterns in each cluster. The distribution of the differential SNPs identified between the cases in a cluster, once the cases had been positioned in a genomic network, was analysed. Moreover, since the philosophy of our programme is to intervene to control TB, we were particularly interested in assessing in detail the current situation, justifying why we focused on the last 3.5 years (January 2020−June 2024).

We found that 106 of the 306 cases (34.6%) diagnosed in the last 3.5 years (i.e. new cases) were clustered. As we had genomic data from the clustered cases identified over the last 21 years, we were able to determine that 56.6% of these new cases joined clusters that had already been identified before the last 3 years (i.e. growing clusters). The remaining 43.4% of new entrances involved recent clusters that had not been identified before this period. We then used our evolutionary analysis of genomic networks, to explore why clusters grew. 

The first point we investigated was whether ongoing transmission drove the incorporation of new cases into clusters. We found that while ongoing transmission contributed to expansion in around two-thirds of recent clusters, it did so in roughly only one-third of historical, growing clusters. This indicates that control efforts addressing ongoing transmission in previously identified clusters may have had a positive impact. Nevertheless, this also means that other factors aside from recent transmission might still be driving the expansion of some clusters. 

Genomic network assessments found that newly clustered cases not due to ongoing transmission in 10 clusters were likely driven by reactivation of past exposures. In these clusters, new cases located in their networks close (0–1 SNPs) to other cases diagnosed ≥ 4 years before. Other studies have also considered as potential reactivations cases linked to an index case in the distant past with few SNP differences between them [15]. Our findings suggest that for these complex, challenging clusters, standard contact tracing in the past may have been suboptimal, with either incomplete identification of all exposed contacts or lack of adherence to prophylactic treatment. Hence, extensive contact tracing and patient close follow-up are important in epidemiologically complex contexts like ours.

We cannot avoid considering the impact that the COVID-19 pandemic may have had on TB dynamics in our study. It could have influenced the proportion of clustered cases that were considered candidates for reactivation. Indeed, an association between COVID-19-related immunosuppression and TB reactivation has been reported, with a suggestion that COVID-19 may accelerate the progression from latent to active TB [16]. We also expected to see a direct effect of the pandemic on TB transmission. Cluster 789 (Figure 1) is an excellent example of a marked asymmetry in the number of secondary cases caused by the same index case and strain before and during the pandemic.

Aside from ongoing transmission and reactivation of past exposures, we found that expansion of clusters may have been caused by cases with prolonged diagnostic delays. In this regard, our analysis of genomic networks identified clusters with a branch connecting two cases diagnosed several years apart, with several SNPs in between. This suggested the involvement of bacterial viability/evolution and therefore the acquisition of diversity prior to diagnosis. As an alternative to our interpretation that the intermediate SNPs could indicate a delay in diagnosis, it has been proposed that diversity could occur during latency with a mutation rate similar to that in active TB [17,18]. The results driving this hypothesis are nevertheless controversial and other studies estimate that the replication rate is lower during latent disease [19]. A recent investigation has highlighted discrepancies in determining the mutation rate during latency [15]. 

In our study, we assessed that new entrances in clusters were likely due to prolonged diagnostic delay (covering the whole period between the new case and the preceding one in the network) if, in the branch connecting two cases, the number of SNPs was as expected for a mutation rate of 1 SNP/2–3 years [20] along the time span between the TB diagnosis of the two cases. Using this approach, we may have overlooked some cases in whom the delay may not be so long and with a lower number of intermediate SNPs in the branch involved. In fact, we did observe examples of possible cases with shorter diagnostic delays which had small numbers of SNPs (2–3 SNPs) in the branches connecting them with their preceding cases. For some of these candidates for shorter diagnostic delays, epidemiological and/or clinical investigations further revealed findings consistent with these, just like for cases with longer diagnostic delays. Nevertheless, it is also possible that the bacterial evolution leading to the higher-than-expected diversity between new clustered cases and the preceding cases identified in some of these clusters may have occurred in other undiagnosed intermediate cases that participated in the cluster but remain undiagnosed.

In reviewing the clinical charts for evidence of undiagnosed TB in the cases proposed as candidates for delayed diagnosis/subclinical TB, we found that FQ-mono-R could also be used as a proxy to consider them, especially when identified in only one member of a cluster. Patients with TB with a previous FQ prescription at TB diagnosis, usually for treatment of community-acquired pneumonia, have a threefold higher risk of having FQ-resistant TB [21]. Multiple FQ prescriptions, FQ prescription more than 60 days prior to TB diagnosis and FQ prescription for more than 10 days are associated with FQ-mono-R [22,23].

Several studies have highlighted the role of subclinical TB in transmission to secondary cases [12,24]. We have also evaluated this and found that the epidemiological circumstances of each case of prolonged diagnostic delay/subclinical TB are critical and may lead to a wide range of effects on the emergence of secondary cases.

This study has some limitations worth considering. The initial screening step to rapidly differentiate between orphan and potentially clustered cases was performed by using MIRU-VNTR genotyping directly on clinical specimens. Although this technique is a useful tool for ruling out clustered cases, we must acknowledge its limited sensitivity for detecting mixed infections. In this regard, it is possible that, if minority variants involved in mixed infections were part of a cluster, these could have gone undetected. Nevertheless, we would expect mixed infections in our population to be anecdotal.

Moreover, all the interpretations derived from the study of the genomic networks of the clusters only led us to propose the involvement of reactivations, diagnostic delay/subclinical TB in these clusters. We cannot be completely sure that the cases who have been analysed because they entered recently in these clusters are exactly those due to reactivations, diagnostic delays/subclinical TB. It is also possible that reactivations, diagnostic delays/subclinical TB corresponded to other previous cases that have recently transmitted the infection to the cases observed in the network and that these potential previous cases were not included in the network, as they were missed because they were not sequenced or even not diagnosed. However, even in this alternative scenario, reactivations, diagnostic delays/subclinical TB would be associated to the growing of the corresponding clusters. We are fairly confident that our analytical coverage is high; our programme has been running since 2003, covering the whole population of Almería, and the figures for the percentage of diagnosed TB cases with culture and sequence available are high (95% of culture-positive cases in the study period could be classified as either orphan or cluster). However, in a migrant-rich population such as ours, the interterritorial mobility of these individuals still leads us to acknowledge the possibility of missed cases.

For this reason, when genomic networks investigations lead us to suspect that new clustered cases are owed to reactivations or delays, additional clinical/epidemiological data are needed to confirm this. It is therefore essential to couple our refined genomic analysis with an equally refined analysis of the clinical and epidemiological data from each new clustered case. This would not be possible without the intervention of our multidisciplinary team, in which members discuss each new case together and re-interview the cases guided by the genomic data, looking for additional information to verify hypotheses. This allowed, in a subset of cases, to find clinical/epidemiological support for our genomic-based classifications.

Identifying the likely involvement of reactivations or diagnostic delays in our clusters, especially when clinical/epidemiological and genomic data concur, allows us to design and propose intervention strategies tailored to the true nature of each clustered case, that can be added to the standard control measures systematically applied to each cluster. For example, when reactivations are validated, the future emergence of secondary cases can be minimised by optimising the preventive treatment of latent TB infection in the context of these cases. In the event of delayed diagnosis, the epidemiological intervention should also include a more active search for potential secondary cases that may have occurred during the time the patient was still undiagnosed. In some clusters we detect independent entries in different branches/locations of the network, which have different possible explanations; this has led us to recommend interventions that are not systematic for a cluster, but rather case specific.

Having understood the usefulness of our deeper use of genomic data, the next steps are to make the results of our approach timelier, so that it can be integrated with the epidemiological investigation in real time. The general approach to genomic analysis in TB is to follow a high-throughput scheme, which means to wait for the accumulation of a large number of isolates to be processed together in the same sequencing run to lower costs. Since the end of our study presented here, we have been using a faster method based on single case nanopore sequencing as soon as the primary culture of each new case is available [25]. The transition from batched strategy to a case-by-case immediate analysis offers an alternative scheme adapted to the early coupling of genomic, epidemiological and clinical data until we can address the fastest approach, sequencing directly on respiratory specimens, which remains pending due to analytical challenges due to the presence of interfering human/bacterial DNA in respiratory samples [26,27].

## Conclusion

We used an evolutionary analysis of the differential SNPs observed in each TB genomic cluster to gain an improved knowledge of the singularities behind each new clustered case. Together with clinical and epidemiological data, this allowed us to more accurately distinguish new cases due to ongoing transmission from new cases due to reactivation of past exposures or new cases that experienced diagnostic delays. Employing this type of integrated analysis will help shed more light on the hidden impact of long delays in diagnosis or subclinical TB. All this information can be of paramount importance for understanding why TB persists in complex populations and for tailoring specific interventions to maximise the success of TB control.

## Data Availability

The bioinformatic pipeline developed and applied and for the genomic analysis is accessible at Git-Hub: https://github.com/MG-IiSGM/autosnippy. The data supporting the results of this study (FASTQ files) were deposited in the ENA (https://www.ebi.ac.uk) under project accession number PRJEB89084.

## Supplementary Data

148000 Supplementary Table1

## Supplementary Data

357000 Supplementary Table2

## Supplementary Data

523000 Supplementary Figure1

## Supplementary Data

361000 Supplementary Figure2
