# Supplementary material for: Long-term refined genomic analysis of tuberculosis clusters to distinguish between ongoing transmission, reactivations or diagnostic delays, Almería, Spain, 2003 to 2024
Source: Euro Surveill. 2026 Mar 19;31(11):2500301. doi: 10.2807/1560-7917.ES.2026.31.11.2500301 (PMC13074182; doi:10.2807/1560-7917.ES.2026.31.11.2500301)
Supplement: Supplementary Table1 [file 25-00301_Supplementary_Table_1.pdf]

This supplementary material is hosted by Eurosurveillance as supporting information alongside the article *Long-term refined genomic analysis of tuberculosis clusters to distinguish between ongoing transmission, reactivations or diagnostic delays*, on behalf of the authors, who remain responsible for the accuracy and appropriateness of the content. The same standards for ethics, copyright, attributions and permissions as for the article apply. Supplements are not edited by Eurosurveillance and the journal is not responsible for the maintenance of any links or email addresses provided therein.

Supplementary Table 1. Lineage identification. The lineages were extracted from the sequences of each strain using the annotation of lineage and sublineage markers obtained from TB-profiler (1) through an in-house script within autosnippy pipeline.

Cluster	Lineage	
2819	4.3	
3201	4.3.4.2	
3330	4.1.2	
3133	4.3.2	
2713	4.3.2	
2778	4.1.1.1	
2964	4.1.2	
2540	4.3.1	
3176	4.8	
3084	4.1.2.1	Recent clusters
3068	4.8	
2661	4.3	
3113	4.1.2.1	
3151	4.8	
1384	4.1.2.1	
3083	4.1.2.1	
2261	4.1.2.1	
2747	4.1.2.1	
2410	4.3.2	
2907	4.1.2.1	
1101	4.1.2.1	
2280	4.6.2.2	
2433	4.8	
2689	2.2.1	
1484	4.1.2.1	
771	4.8	
786	4.1.2.1	
1330	4.1.2.1	
143	4.3.2	
1482	4.3.2	
1202	4.4.1.1	
1180	4.3.3	
106	4.4.1.1	
493	4.1.2.1	
347	4.3.4.2	Growing clusters
778	4.1.2.1	
1304	4.1.2.1	
30	4.8	
60	4.1.2.1	
15	4.3.3	
680	4.1.2.1	
789	4.8	
558	4.3.2	
630	4.1.2.1	
76	4.1.2.1	
1338	4.8	
535	4.1.2.1	
2581	4.8	
2458	3	
386	4.3.2	
1803	4.4.1.1	
2054	4.3.2	
1566	4.3.2	

ified as ECDC NORMAL

1. Phelan JE, O'Sullivan DM, Machado D, Ramos J, Oppong YEA, Campino S, O'Grady J, McNerney R, Hibberd ML, Viveiros M, Huggett JF, Clark TG. 2019. Integrating informatics tools and portable sequencing technology for rapid detection of resistance to anti-tuberculous drugs. Genome Med 11:1–7.
