# Supplementary material for: Long-term refined genomic analysis of tuberculosis clusters to distinguish between ongoing transmission, reactivations or diagnostic delays, Almería, Spain, 2003 to 2024
Source: Euro Surveill. 2026 Mar 19;31(11):2500301. doi: 10.2807/1560-7917.ES.2026.31.11.2500301 (PMC13074182; doi:10.2807/1560-7917.ES.2026.31.11.2500301)
Supplement: Supplementary Table2 [file 25-00301_Supplementary_Table_2.pdf]

This supplementary material is hosted by Eurosurveillance as supporting information alongside the article *Long-term refined genomic analysis of tuberculosis clusters to distinguish between ongoing transmission, reactivations or diagnostic delays*, on behalf of the authors, who remain responsible for the accuracy and appropriateness of the content. The same standards for ethics, copyright, attributions and permissions as for the article apply. Supplements are not edited by Eurosurveillance and the journal is not responsible for the maintenance of any links or email addresses provided therein.

**Supplementary Table 2 |** Socio-demographic data for cases in cluster

| Clusters | Patient ID | Country of origin | District | Health area |
|---|---|---|---|---|
| 2819 | p2819 | Morocco | West | Adra |
| | p2831 | Spain (Morocco)* | West | Adra |
| 3201 | p3201 | Morocco | Levante-Alto Almanzora | Garrucha |
| | p3259 | Morocco | Levante-Alto Almanzora | Vera |
| 3330 | p3330 | Morocco | Almeria | San Isidro De Nijar |
| | p3335 | Morocco | Almeria | San Isidro De Nijar |
| 3133 | p3133 | Morocco | West | La Mojonera |
| | p3205 | Morocco | West | La Mojonera Venta del Viso |
| | p3283 | Morocco | West | La Mojonera |
| | p3300 | Mali | West | San Agustin |
| 2713 | p2713 | Morocco | West | El Ejido |
| | p2993 | Morocco | West | La Mojonera |
| | p2996 | Spain (Morocco)* | West | La Mojonera |
| 2778 | p2778 | Senegal | Almeria | Carboneras |
| | p3157 | Senegal | Almeria | Carboneras |
| 2964 | p2964 | Morocco | Almeria | Saladar Y Leche |
| | p3281 | Romaleia | Almeria | Nijar |
| 2540 | p2540 | Morocco | Almeria | San Isidro De Nijar |
| | p2989 | Morocco | Almeria | Campohermoso |
| | p3105 | Morocco | Almeria | Campohermoso |
| 3176 | p3176 | Morocco | West | El Ejido-Las Norias de Daza |
| | p3218 | Spain (Morocco)* | West | Vícar-Puebla de Vícar |
| 3084 | p3084 | Morocco | West | Roquetas De Mar |
| | p3244 | Morocco | West | El Ejido-Santa María del Águila |
| 3068 | p3068 | Spain | Almeria | Tahal |
| | p3223 | Romaleia | Almeria | Tahal |
| 2661 | p2661 | Morocco | West | Vícar-Puebla de Vícar |
| | p3192 | Morocco | West | Vícar-Gangosa |
| 3113 | p3113 | Guinea Bissau | Levante-Alto Almanzora | Turre |

| | | | | |
|---|---|---|---|---|
| | p3217 | Guinea Bissau | Levante-Alto Almanzora | Turre |
| | p3115 | Guinea Bissau | West | El Ejido-Las Norias de Daza |
| | p3121 | Gambia | West | Roquetas De Mar |
| | p3139 | Burkina Faso | West | La Mojonera |
| | p3306 | Spain (Guinea Bissau, Senegal)* | West | Roquetas De Mar |
| 3151 | p3151 | Spain | Levante-Alto Almanzora | Vera |
| | p3285 | Spain | Levante-Alto Almanzora | Arboleas |
| 1384 | p1348 | Morocco | West | Vícar-Puebla de Vícar |
| | p3142 | Morocco | West | El Ejido-Santa María del Águila |
| 3083 | p3083 | Morocco | West | Berja |
| | p3087 | Senegal | West | Berja |
| | p3185 | Morocco | West | Berja |
| | p3272 | Morocco | West | Berja |
| 2261 | p2261 | Morocco | West | El Ejido-Santa María del Águila |
| | p3067 | Morocco | Levante-Alto Almanzora | San Isidro De Nijar |
| 2747 | p2747 | Morocco | Levante-Alto Almanzora | Fines |
| | p3198 | Senegal | West | Roquetas De Mar |
| 2410 | p2410 | Morocco | West | Vícar-Gangosa |
| | p3222 | Morocco | West | El Ejido-Guardias Viejas |
| 2907 | p2907 | Mali | West | El Ejido |
| | p2823 | Mali | West | El Ejido |
| | p2957 | Senegal | West | El Ejido |
| 1101 | p1101 | Romaleia | West | Roquetas De Mar |
| | p3275 | Romaleia | Almeria | Almeria-Retamar |
| 2280 | p2280 | Equatorial Guinea | West | Roquetas de Mar (Cortijos de Marín) |
| | p3094 | Guinea Bissau | West | Berja |
| | p3097 | United Kingdom | West | El Ejido |
| 2433 | p2433 | Spain | Almeria | Cuevas de los Medinas (Prisión) |
| | p2539 | Romaleia | Almeria | Cuevas de los Medinas (Prisión) |
| | p2616 | Algeria | Almeria | Cuevas de los Medinas (Prisión) |
| | p2584 | Morocco | Almeria | Cuevas de los Medinas (Prisión) |
| | p2838 | Spain | Almeria | Cuevas de los Medinas (Prisión) |

Classified as ECDC NORMAL

| | | | | |
|---|---|---|---|---|
| 2689 | p2689 | Ukraine | West | Adra |
| | p2759 | Spain | West | Adra |
| | p2812 | Spain | West | Balanegra |
| | p2822 | Spain | West | Adra |
| 1484 | p1484 | Morocco | West | La Mojonera |
| | p2351 | Morocco | West | La Mojonera |
| | p2359 | Morocco | West | La Mojonera |
| | p2516 | Morocco | West | El Ejido |
| | p3005 | Senegal | West | Vícar-Puebla de Vícar |
| 771 | p771 | Morocco | Almeria | Almeria |
| | p1782 | Spain | Almeria | Almería 04005 |
| | p1540 | Morocco | Almeria | Almería-La Cañada |
| | p2345 | Spain | Almeria | Almería 04008 |
| | p2788 | Spain | Almeria | Almería 04007 |
| | p1788 | Spain | Almeria | Tijola |
| | p2731 | Spain | Almeria | Carboneras |
| | p2757 | Spain | Almeria | Carboneras |
| 786 | p786 | Romaleia | Almeria | Almeria |
| | p1228 | Romaleia | Almeria | Almería 04007 |
| | p797 | Romaleia | Almeria | Almería 04005 |
| | p2642 | Romaleia | Almeria | Almería 04009 |
| | p2776 | Spain | Almeria | Almería 04003 |
| | p2892 | Spain | Almeria | Almería 04009 |
| | p1813 | Spain | Almeria | Almería 04007 |
| | p2682 | Morocco | Almeria | Almería 04009 |
| | p2729 | Morocco | Almeria | Almería 04009 |
| 1330 | p1330 | Spain | West | Berja |
| | p1694 | Spain | West | Laujar De Andarax |
| | p1906 | Spain | West | Berja |
| | p1603 | Spain | Almeria | Nijar |
| | p2800 | Spain | West | Berja |
| | p1480 | Spain | West | Berja |
| | p1772 | Spain | West | Berja |
| 143 | p143 | Spain | Another Province | Otra Provincia |
| | p2327 | Spain | Levante-Alto Almanzora | Tijola |
| | p3081 | Spain | Almeria | Almería 04001 |
| | p2032 | Spain | Almeria | Almería 04007 |
| | p824 | Spain | Levante-Alto Almanzora | Purchena |
| | p1660 | Ecuador | Almeria | Almería 04008 |

| | | | | |
|---|---|---|---|---|
| | p2397 | Spain | Levante-Alto Almanzora | Purchena |
| 1482 | p1482 | Morocco | Almeria | Atochares |
| | p1874 | Mali | Almeria | San Isidro De Nijar |
| | p1821 | Morocco | Almeria | San Isidro De Nijar |
| | p2008 | Morocco | Almeria | San Isidro De Nijar |
| | p2042 | Morocco | Almeria | San Isidro De Nijar |
| | p2143 | Morocco | Almeria | El Viso |
| | p2201 | Spain | Almeria | San Isidro De Nijar |
| | p2480 | Morocco | Almeria | Campohermoso |
| | p3166 | Spain | Almeria | Nijar |
| | p2251 | Nigeria | Levante-Alto Almanzora | Vera |
| 1202 | p1202 | Senegal | West | El Ejido-Santa María del Águila |
| | p1469 | Senegal | West | Roquetas De Mar |
| | p1281 | Morocco | West | Berja |
| | p1498 | Senegal | West | Venta Del Viso |
| | p1733 | Senegal | West | Roquetas De Mar |
| | p1965 | Senegal | West | Balanegra |
| | p2023 | Spain | West | Balanegra |
| | p2029 | Senegal | West | El Ejido |
| | p2553 | Gambia | West | Roquetas De Mar |
| | p1995 | Senegal | West | Balanegra |
| | p3149 | Morocco | West | Balanegra |
| | p2430 | Senegal | Almeria | Almería 04008 |
| 1180 | p1180 | Spain | Almeria | Almería 04006 |
| | p2080 | Morocco | West | Vícar-Cabañuelas |
| | p2120 | Morocco | West | Roquetas De Mar |
| | p2155 | Argentina | Almeria | Almería 04007 |
| | p2167 | Argentina | Almeria | Almería 04007 |
| | p2532 | Spain | Almeria | Almería 04009 |
| | p2165 | Argentina | Almeria | Almería 04004 |
| | p2295 | Spain | Almeria | Almería 04006 |
| | p3184 | Spain | Almeria | Almería 04007 |
| 106 | p3245 | Spain | West | Roquetas De Mar |
| | p109 | Spain | West | Roquetas De Mar |
| | p1856 | Spain | Levante-Alto Almanzora | Vera |
| | p419 | Spain | Levante-Alto Almanzora | Macael |
| 493 | p493 | Morocco | West | El Ejido-Las Norias de Daza |
| | p523 | Morocco | West | El Ejido-Las Norias de Daza |
| | p3153 | Morocco | West | El Ejido-Las Norias de Daza |

| | | | | |
|---|---|---|---|---|
| | p576 | Morocco | West | El Ejido-Las Norias de Daza |
| | p2249 | Equatorial Guinea | West | La Mojonera |
| | p2368 | Spain | West | Roquetas De Mar |
| | p2721 | Spain | West | Roquetas De Mar |
| | p2286 | Morocco | West | El Ejido-Santa María del Águila |
| | p2442 | Mauritania | West | Roquetas De Mar |
| | p3236 | Senegal | West | El Ejido |
| | p2463 | Morocco | West | Vícar-Gangosa |
| 347 | p347 | Mauritania | West | Roquetas De Mar |
| | p571 | Mauritania | West | Cortijada De Marin |
| | p1349 | Senegal | West | Vicar |
| | p3239 | Mali | West | Roquetas De Mar |
| | p2098 | Senegal | West | Balanegra |
| | p3329 | Senegal | West | Roquetas De Mar |
| | p3042 | Senegal | West | Roquetas De Mar |
| | p550 | Mauritania | West | Vicar |
| | p2490 | Senegal | West | Roquetas De Mar |
| | p2519 | Spain | West | Aguadulce |
| | p3145 | Mali | West | Roquetas De Mar |
| | p2468 | Senegal | West | Vícar-Canal (La) |
| | p3268 | Senegal | West | Vícar-Gangosa |
| 778 | p778 | Algeria | West | El Ejido |
| | p1497 | Spain | West | El Ejido |
| | p3158 | Algeria | Levante-Alto Almanzora | Cuevas De Almanzora |
| 1304 | p1304 | Spain | Almeria | El Alquian |
| | p1863 | Spain | Almeria | Almería 04003 |
| | p1599 | Spain | Almeria | Almería 04009 |
| | p1796 | Spain | Almeria | Almería-La Cañada |
| | p2310 | Spain | West | Roquetas de Mar (El Parador de las Hortichuelas) |
| | p1558 | Spain | West | El Ejido-Balerma |
| | p2003 | Spain | West | Berja |
| | p2873 | Spain | West | El Ejido-Santa María del Águila |
| | p3072 | Spain | Almeria | Almería 04007 |
| 30 | p30 | Spain | West | Roquetas De Mar |
| | p1512 | Morocco | West | Roquetas de Mar (Aguadulce) |
| | p1525 | Spain | West | Roquetas De Mar |
| | p3219 | Slovakia | West | Roquetas De Mar |
| | p793 | Spain | West | Roquetas De Mar |

| | | | | |
|---|---|---|---|---|
| | p1473 | Spain | West | Roquetas De Mar |
| | p2039 | Spain | West | Roquetas De Mar |
| | p549 | Spain | West | Roquetas De Mar |
| | p655 | Spain | West | Roquetas De Mar |
| | p679 | Senegal | West | El Ejido-Las Norias de Daza |
| | p990 | Nigeria | West | Roquetas De Mar |
| | p1513 | Spain | West | Roquetas De Mar |
| 60 | p60 | Spain | West | Adra |
| | p450 | Spain | Almeria | La Cañada De San Urbano |
| | p450 | Spain | Almeria | La Cañada De San Urbano |
| | p3180 | Spain | West | Adra |
| 15 | p15 | Romania | Levante-Alto Almanzora | Garrucha |
| | p392 | Spain | Levante-Alto Almanzora | Mojacar |
| | p563 | Spain | Levante-Alto Almanzora | Vera |
| | p2874 | Spain | Levante-Alto Almanzora | Vera |
| | p3161 | Spain | Levante-Alto Almanzora | Vera |
| | p405 | Spain | Levante-Alto Almanzora | Garrucha |
| | p661 | Spain | Levante-Alto Almanzora | Vera |
| | p1080 | Spain | Levante-Alto Almanzora | Antas |
| | p1152 | Spain | Levante-Alto Almanzora | Mojacar |
| | p1939 | Spain | Levante-Alto Almanzora | Vera |
| | p1977 | Spain | Levante-Alto Almanzora | Cuevas De Almanzora |
| | p2224 | Spain | Levante-Alto Almanzora | Cuevas De Almanzora |
| | p2290 | Spain | Almeria | Almería 04004 |
| | p2621 | Spain | Levante-Alto Almanzora | Vera |
| | p2705 | Spain | Almeria | Almería 04006 |
| | p3034 | Spain | Levante-Alto Almanzora | Antas |
| | p2427 | Bolivia | Levante-Alto Almanzora | Antas |
| | p2427 | Bolivia | Levante-Alto Almanzora | Antas |
| 680 | p680 | Romania | Almeria | Tabernas |
| | p1233 | Romania | Almeria | Tabernas |
| | p2833 | Spain | Almeria | Tabernas |
| | p1252 | Spain (Romania)* | Almeria | Tabernas |
| | p3173 | Spain | Almeria | Tabernas |
| 789 | p789 | Mali | West | Vícar-Yegua Verde-El Congo |
| | p1663 | Nigeria | West | El Ejido |
| | p2085 | Mali | West | Roquetas de Mar (Cortijos de Marín) |
| | p2418 | Mali | West | Roquetas de Mar (Cortijos de Marín) |

| | | | | |
|---|---|---|---|---|
| | p1761 | Mali | West | La Mojonera |
| | p1838 | Mali | West | Vícar-Llanos de Vícar |
| | p3292 | Mali | West | La Mojonera |
| | p3204 | Spain | West | Roquetas De Mar |
| | p2536 | Gambia | West | Roquetas de Mar (Cortijos de Marín) |
| | p2570 | Mali | West | Roquetas de Mar (Cortijos de Marín) |
| | p2678 | Mali | West | La Mojonera |
| | p2939 | Mali | West | Vícar-Yegua Verde-El Congo |
| | p1883 | Mali | West | Roquetas de Mar (Cortijos de Marín) |
| | p2534 | Mali | West | Roquetas De Mar |
| | p2534 | Mali | West | Roquetas De Mar |
| | p2816 | Mali | West | Roquetas De Mar |
| | p2651 | Mali | West | Roquetas De Mar |
| | p2712 | Spain | West | Roquetas De Mar |
| | p2745 | Spain | West | Vícar-Gangosa |
| | p2961 | Mali | West | Roquetas De Mar |
| | p3025 | Senegal | West | Roquetas De Mar |
| | p3085 | Mali | West | Puebla De Vicar |
| | p3089 | Mali | West | Vícar-Llanos de Vícar |
| | p3137 | Burkina Faso | West | Roquetas De Mar |
| | p3179 | Mali | West | Vícar-Llanos de Vícar |
| | p3030 | Morocco | West | Roquetas De Mar |
| 558 | p558 | Morocco | West | El Ejido-Las Norias de Daza |
| | p1843 | Morocco | West | El Ejido-San Agustín |
| | p756 | Morocco | West | El Ejido-Matagorda |
| | p1429 | Morocco | West | El Ejido-Tarambana |
| | p3002 | Spain | West | Vícar-Archilla-Cañada Sebastiana |
| | p752 | Morocco | West | Cortijada De Marin |
| | p2252 | Morocco | West | El Ejido-Matagorda |
| | p3175 | Spain | West | Roquetas De Mar |
| | p3215 | Morocco | West | Cuevas de los Medinas (Prisión) |
| | p3159 | Morocco | West | Roquetas De Mar |
| | p3315 | Morocco | West | El Ejido |
| 630 | p630 | Romania | Levante-Alto Almanzora | Huercal-Overa |
| | p630 | Romania | Levante-Alto Almanzora | Huercal-Overa |
| | p912 | Romania | Levante-Alto Almanzora | Huercal-Overa |
| | p3318 | Colombia | Levante-Alto Almanzora | Huercal-Overa |

| | | | | |
|---|---|---|---|---|
| | p3252 | Spain | Levante-Alto Almanzora | El Saltador |
| 76 | p76 | Spain | Almeria | Almería 04009 |
| | p156 | Spain | Almeria | Almería 04001 |
| | p681 | Spain | Almeria | Almeria |
| | p1740 | Spain | Almeria | El Barranquete |
| | p3282 | Spain | Almeria | Almería 04008 |
| | p416 | Spain | West | Adra |
| | p1683 | Spain | Almeria | Almería 04002 |
| | p1847 | Spain | Almeria | Almería 04003 |
| | p2028 | Spain | Almeria | Almería 04009 |
| | p2425 | Spain | Almeria | Almería 04008 |
| | p510 | Spain | Almeria | Almeria |
| | p512 | Spain | Levante-Alto Almanzora | Antas |
| | p822 | Spain | Almeria | Almería 04003 |
| | p838 | Spain | Almeria | Almeria |
| | p1486 | Spain | Almeria | Almería 04006 |
| | p2200 | Spain | Almeria | Almería 04007 |
| | p2278 | Spain | Almeria | Almería 04008 |
| | p3130 | Spain | West | Roquetas De Mar |
| | p3125 | Spain | West | El Ejido |
| 1338 | p1338 | Lithuania | Almeria | El Viso |
| | p1570 | China | Almeria | Campohermoso |
| | p3075 | Spain | Almeria | Pechina |
| 535 | p535 | Brazil | West | Roquetas de Mar (Aguadulce) |
| | p2109 | Lithuania | Almeria | Almería 04008 |
| | p3262 | Spain | Almeria | Almería 04003 |
| 2581 | p2581 | Morocco | West | La Mojonera |
| | p2675 | Morocco | West | La Mojonera |
| | p3266 | Morocco | West | El Ejido-Las Norias de Daza |
| 2458 | p2458 | Mali | West | Roquetas de Mar (Cortijos de Marín) |
| | p2521 | Senegal | West | Adra |
| | p3163 | Mauritania | West | Roquetas de Mar (Cortijos de Marín) |
| 386 | p386 | Morocco | West | Vicar |
| | p1641 | Morocco | Levante-Alto Almanzora | Pulpi |
| | p1985 | Morocco | West | El Ejido |
| | p3155 | Morocco | Almeria | Gergal |
| | p3209 | Morocco | West | Vícar-Cabañuelas |
| 1803 | p2520 | Morocco | West | El Ejido |

| | | | | |
|---|---|---|---|---|
| | p1803 | Morocco | West | Vícar-Archilla-Cañada Sebastiana |
| | p2785 | Morocco | Almeria | Almería 04008 |
| 2054 | p2054 | Spain (Morocco)* | Almeria | Almería 04002 |
| | p2055 | Morocco | Almeria | Almería 04002 |
| | p3265 | Morocco | West | Roquetas De Mar |
| 1566 | p1566 | Spain | Almeria | Almería 04009 |
| | p260 | Spain | Almeria | Almería 04002 |
| | p271 | Spain | Almeria | Almería 04002 |
| | p545 | Spain | Levante-Alto Almanzora | Huercal-Overa |
| | p2669 | Spain | West | El Ejido |
| | p3096 | Spain | Almeria | Cuevas de los Medinas (Prisión) |

* Samples correspond to patients born in Spain with with family members who immigrated to Spain (nationalities of origin are indicated in brackets).
Median age for the patients is 35 years (ranging from 0-83) divided into 236 male and 65 female patients.
