# Supplementary material for: Long-term refined genomic analysis of tuberculosis clusters to distinguish between ongoing transmission, reactivations or diagnostic delays, Almería, Spain, 2003 to 2024
Source: Euro Surveill. 2026 Mar 19;31(11):2500301. doi: 10.2807/1560-7917.ES.2026.31.11.2500301 (PMC13074182; doi:10.2807/1560-7917.ES.2026.31.11.2500301)
Supplement: Supplementary Figure1 [file 25-00301_Supplementary_Figure_1.pdf]

This supplementary material is hosted by Eurosurveillance as supporting information alongside the article *Long-term refined genomic analysis of tuberculosis clusters to distinguish between ongoing transmission, reactivations or diagnostic delays*, on behalf of the authors, who remain responsible for the accuracy and appropriateness of the content. The same standards for ethics, copyright, attributions and permissions as for the article apply. Supplements are not edited by Eurosurveillance and the journal is not responsible for the maintenance of any links or email addresses provided therein.

Supplementary Figure 1 | Genomic network of the 53 clusters involving new clustered cases diagnosed between 2021 and 2024 (highlighted as shaded). The first number corresponds to the case and the second one to the year of diagnosis. In the genomic networks, black dots represent SNPs, and cases within the same box correspond to identical strains (0 SNPs between them). Clusters or sections of clusters highlighted by bold borders are those included in Figures 1-5. mv: median vectors: correspond to hypothetical nodes in the network that correspond to intermediate cases who participated in the transmission but were missed/non-diagnosed (if these hypothetical nodes are not intermediate in the network but preceded the cases are drawn as a shaded circle)

Recent clusters

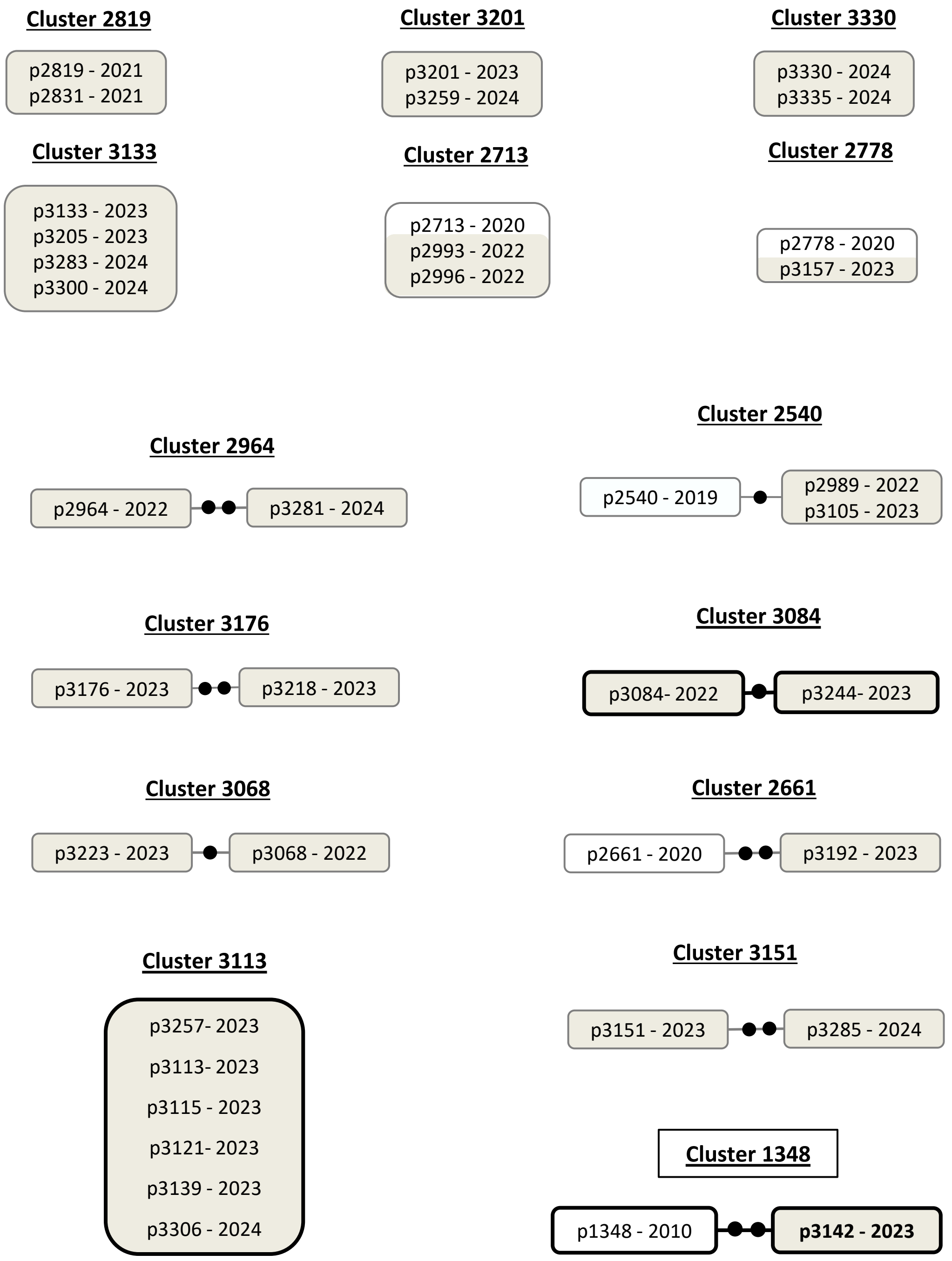

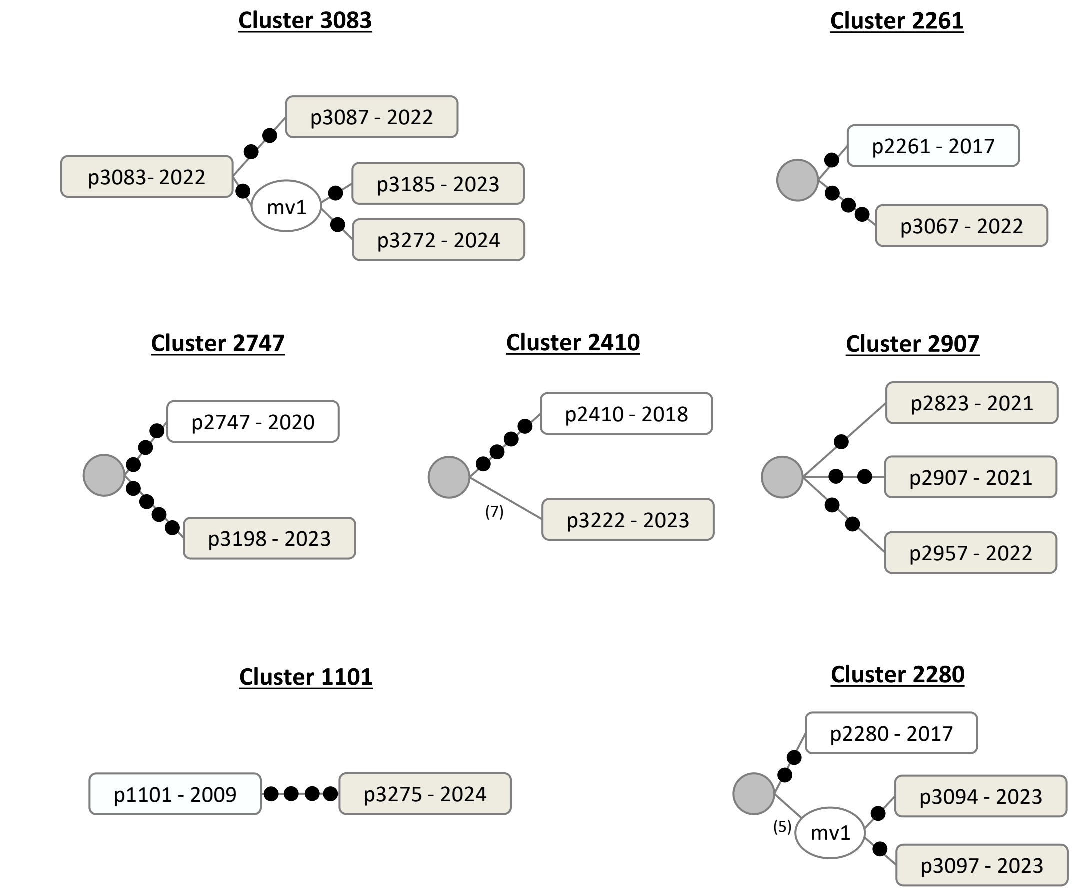

Growing clusters

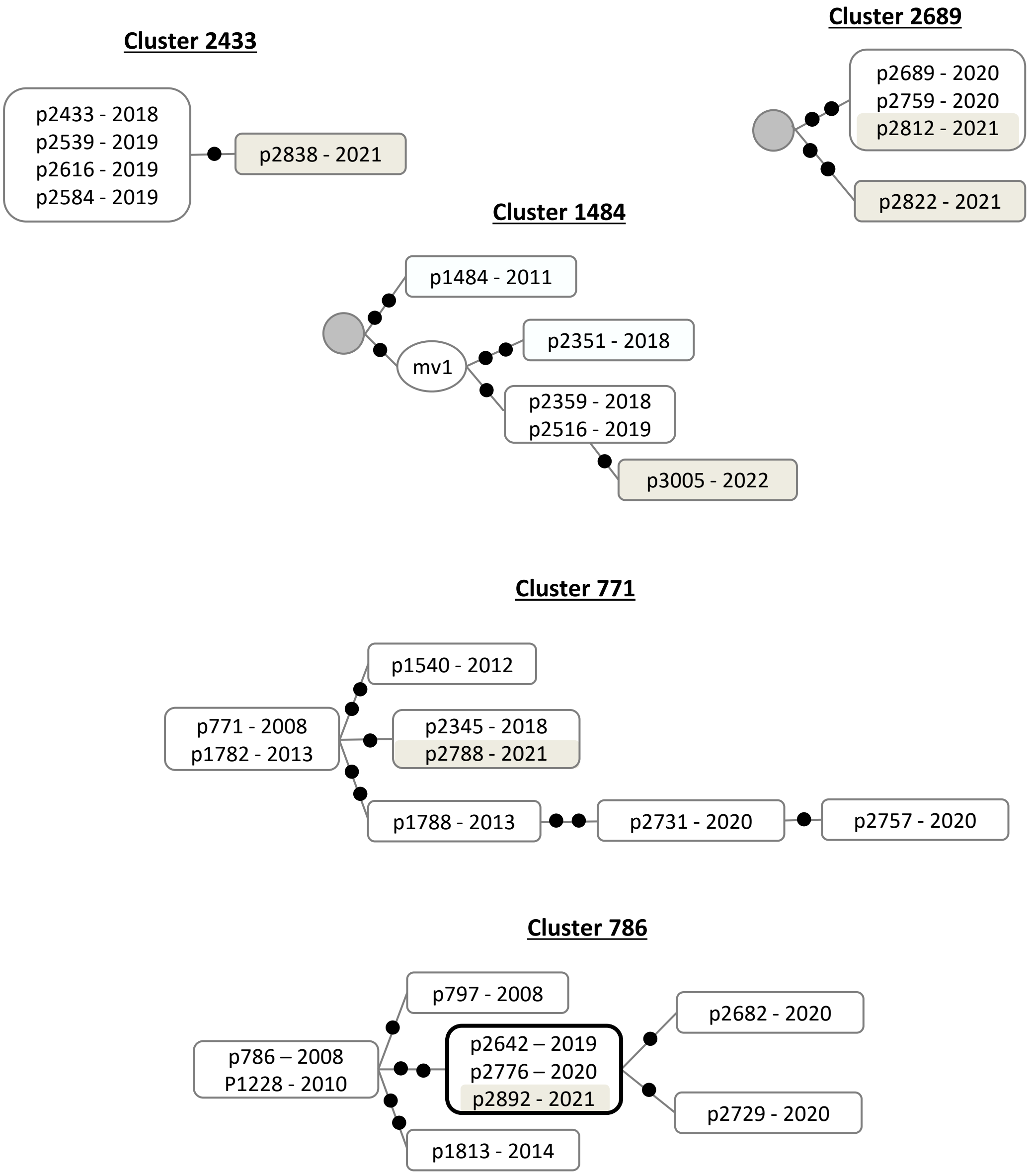

Cluster 1330

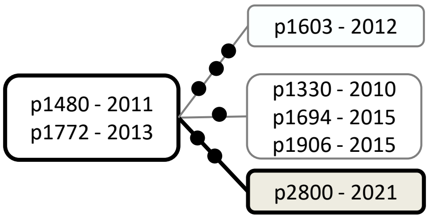

Cluster 143

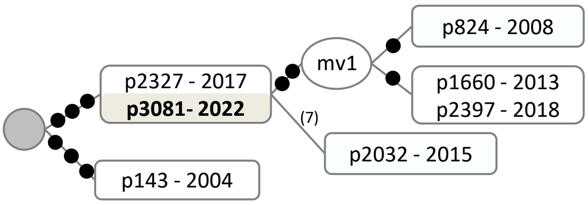

Cluster 1482

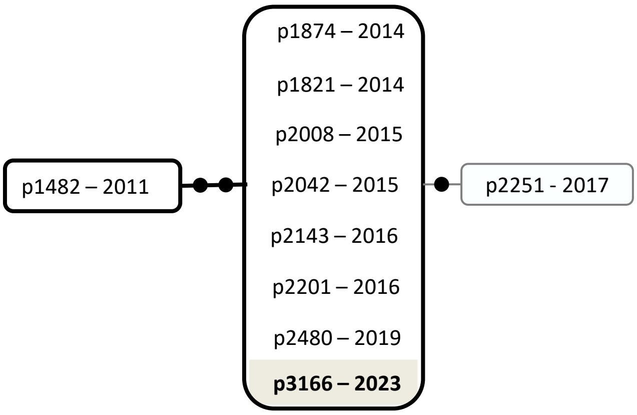

Cluster 1202

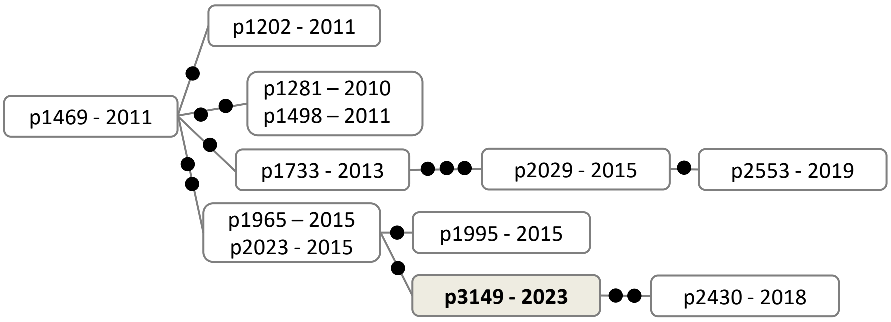

Cluster 1180

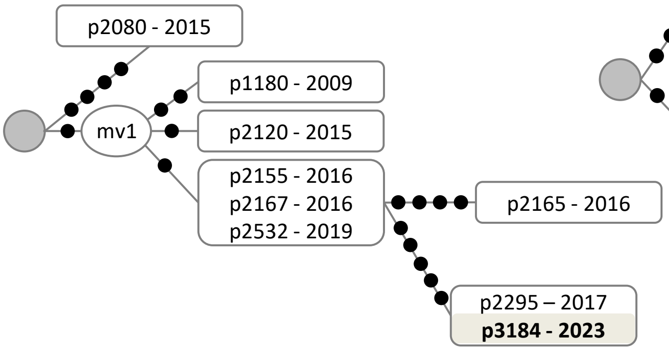

Cluster 106

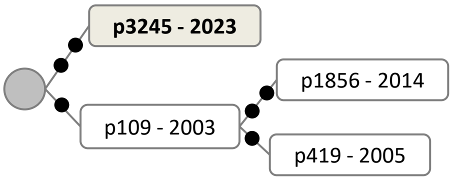

Cluster 493

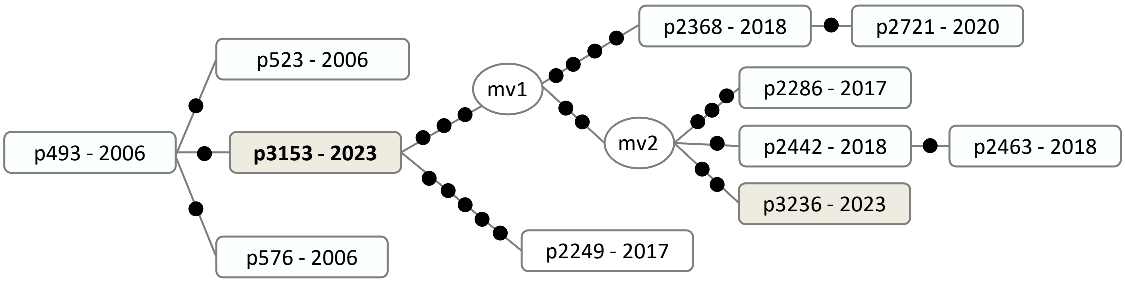

Cluster 347

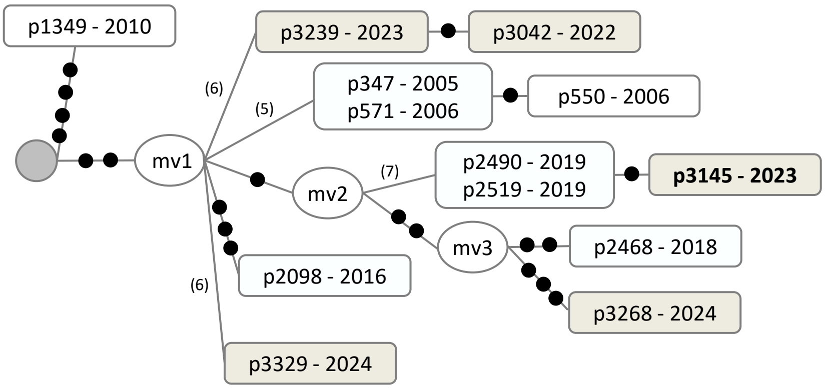

Cluster 778

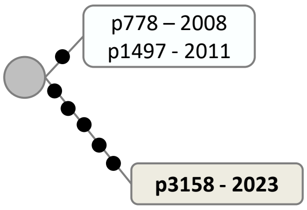

Cluster 1304

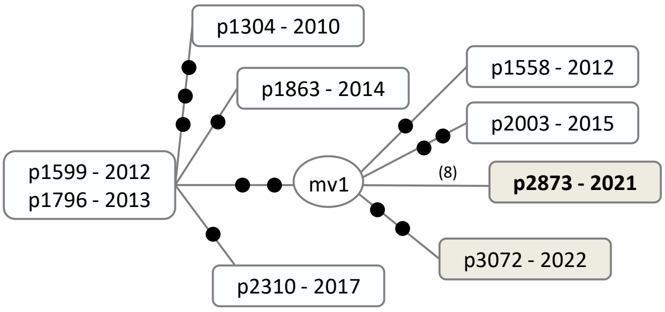

Cluster 30

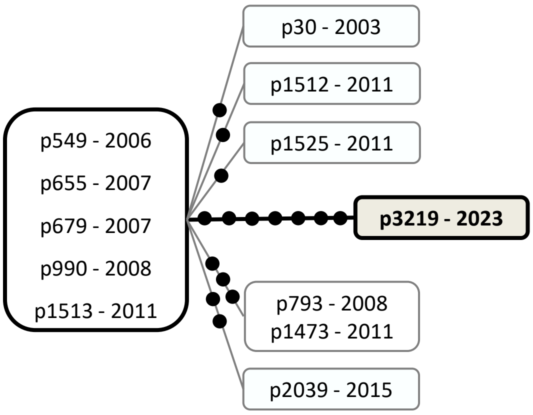

Cluster 60

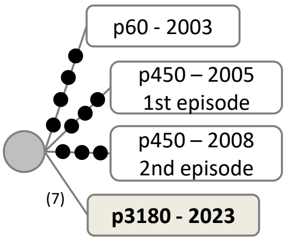

Cluster 15

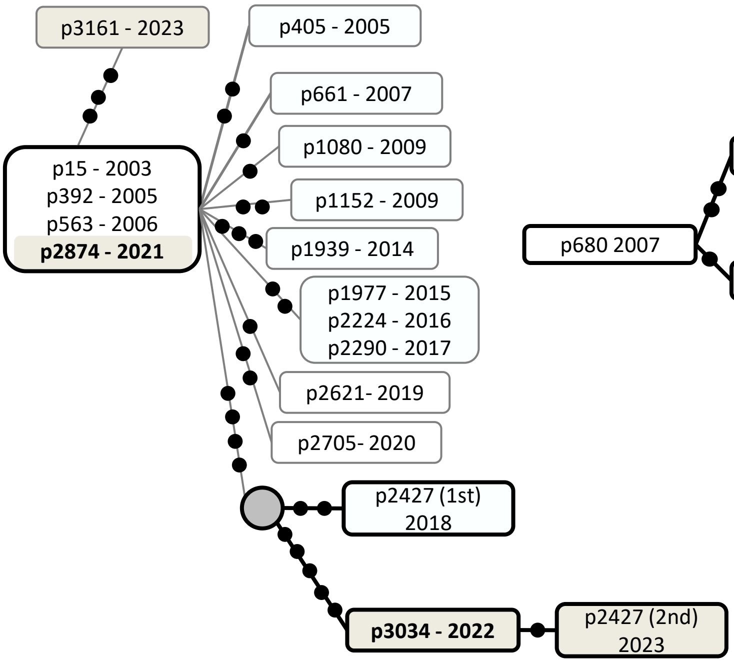

Cluster 680

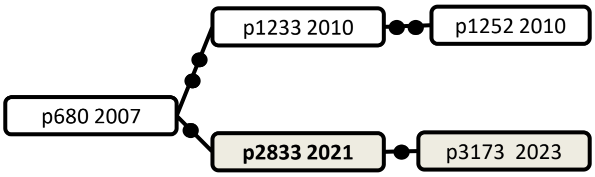

Cluster 789

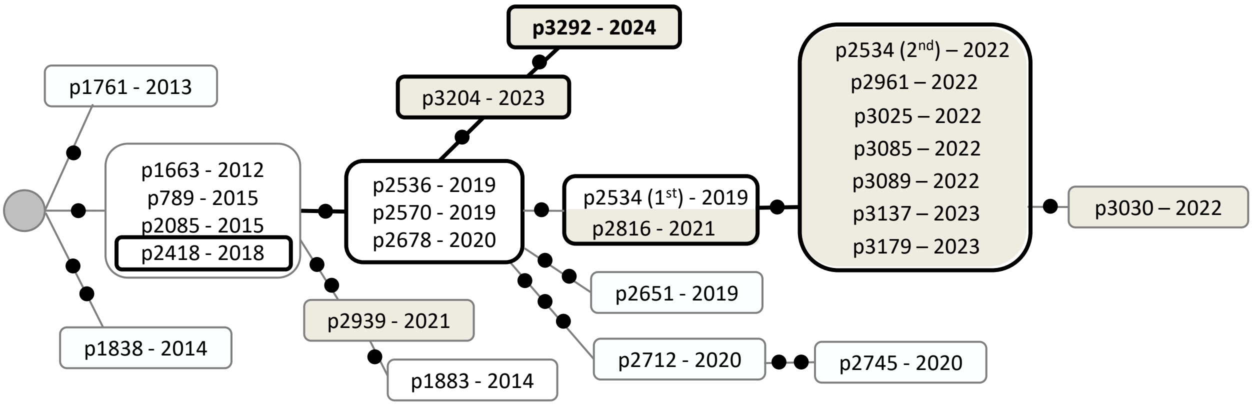

Cluster 558

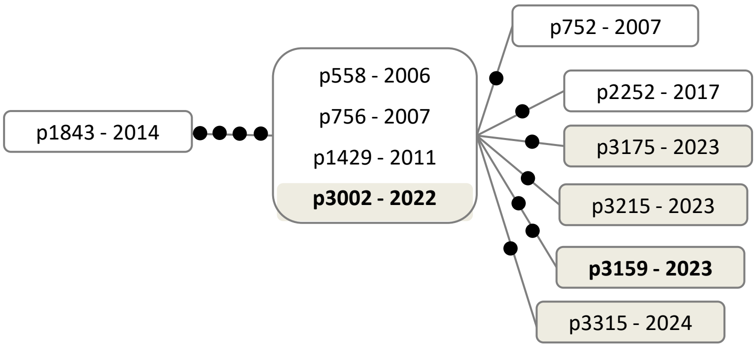

Cluster 630

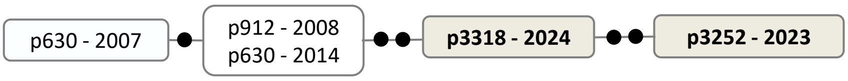

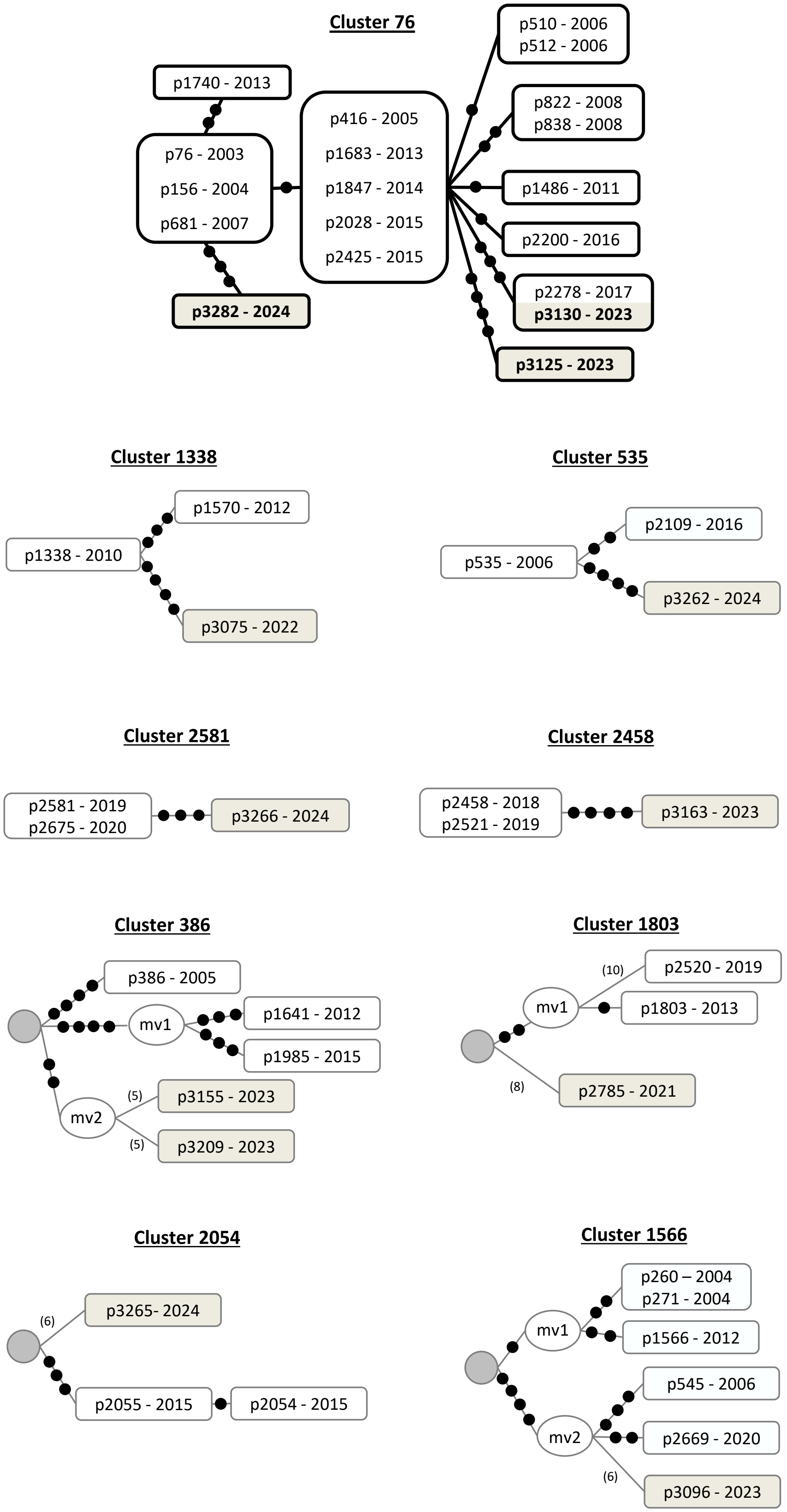
Cluster 76
p1740 - 2013
p76 - 2003
p156 - 2004
p681 - 2007
p3282 - 2024
p416 - 2005
p1683 - 2013
p1847 - 2014
p2028 - 2015
p2425 - 2015
p510 - 2006
p512 - 2006
p822 - 2008
p838 - 2008
p1486 - 2011
p2200 - 2016
p2278 - 2017
p3130 - 2023
p3125 - 2023
Cluster 1338
p1570 - 2012
p1338 - 2010
p3075 - 2022
Cluster 535
p2109 - 2016
p535 - 2006
p3262 - 2024
Cluster 2581
p2581 - 2019
p2675 - 2020
p3266 - 2024
Cluster 2458
p2458 - 2018
p2521 - 2019
p3163 - 2023
Cluster 386
p386 - 2005
mv1
p1641 - 2012
p1985 - 2015
mv2
(5)
p3155 - 2023
(5)
p3209 - 2023
Cluster 1803
(10)
p2520 - 2019
mv1
p1803 - 2013
(8)
p2785 - 2021
Cluster 2054
(6)
p3265- 2024
p2055 - 2015
p2054 - 2015
Cluster 1566
p260 – 2004
p271 - 2004
mv1
p1566 - 2012
p545 - 2006
mv2
p2669 - 2020
(6)
p3096 - 2023
