# Supplementary material for: Long-term refined genomic analysis of tuberculosis clusters to distinguish between ongoing transmission, reactivations or diagnostic delays, Almería, Spain, 2003 to 2024
Source: Euro Surveill. 2026 Mar 19;31(11):2500301. doi: 10.2807/1560-7917.ES.2026.31.11.2500301 (PMC13074182; doi:10.2807/1560-7917.ES.2026.31.11.2500301)
Supplement: Supplementary Figure2 [file 25-00301_Supplementary_Figure_2.pdf]

This supplementary material is hosted by Eurosurveillance as supporting information alongside the article *Long-term refined genomic analysis of tuberculosis clusters to distinguish between ongoing transmission, reactivations or diagnostic delays*, on behalf of the authors, who remain responsible for the accuracy and appropriateness of the content. The same standards for ethics, copyright, attributions and permissions as for the article apply. Supplements are not edited by Eurosurveillance and the journal is not responsible for the maintenance of any links or email addresses provided therein.

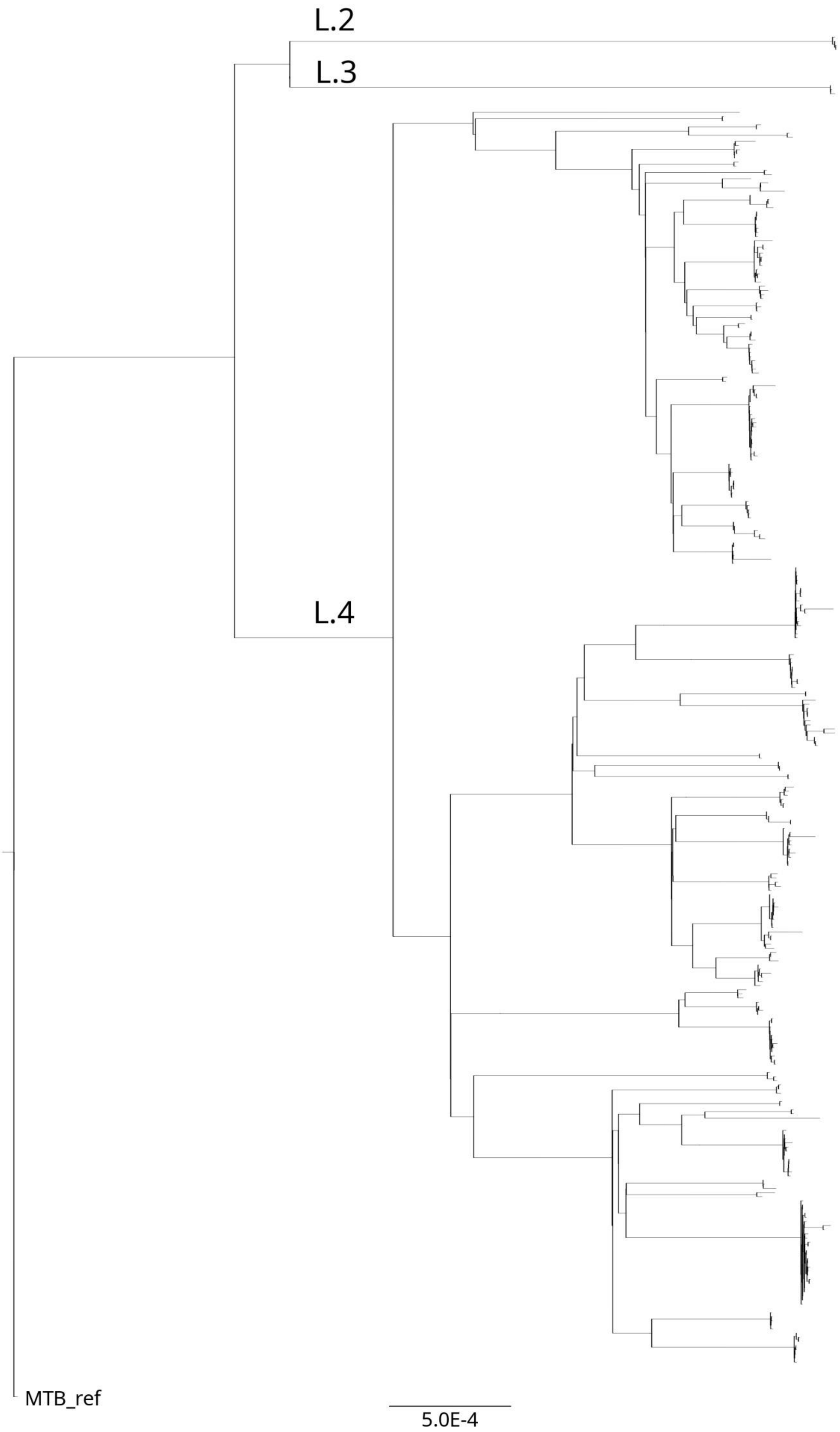

Phylogenetic analysis of the *M. tuberculosis* clusters in the study. Maximum likelihood phylogenetic tree including the sequences from the clusters selected for study (clusters including cases for the 2021-June 2024 period). Scale corresponds to nucleotide divergence (SNP/site). A differential SNP analysis using snippy (included in autosnippy) was performed. Then, a multiple sequence alignment including only variable sites was generated to construct a high-resolution phylogeny. The phylogenetic tree was inferred using RAxML v8.2.12 (Randomized Axelerated Maximum Likelihood) software with the General Time Reversible (GTR) nucleotide substitution model and GAMMA rate heterogeneity with Lewis's ascertainment bias correction for SNP-only data. We performed 1000 bootstrap replicates and 10 independent ML searches applying a seed '12345' to ensure robustness and reproducibility. The final tree was rooted on the ancestral MTB reference genome and visualized using Figtree v1.4.4.
